# Cognitive Dysfunction and Vascular/Valvular Calcification in Patients Undergoing Peritoneal Dialysis: A Cross-Sectional Study

**DOI:** 10.3390/jcm15103635

**Published:** 2026-05-09

**Authors:** Nazife Nur Özer Şensoy, Mehmet Usta, Selvi Coşar, Furkan Ertürk Urfalı, Doğaç Koruk, Süleyman Bekirçavuşoğlu, Türker Emre

**Affiliations:** 1 Department of Nephrology, University of Health Sciences, Bursa Yüksek İhtisas Training and Research Hospital, 16310 Bursa, Türkiye; 2 Department of Nephrology, University of Health Sciences, Bursa City Training and Research Hospital, 16250 Bursa, Türkiye; dr.mehmet.usta@hotmail.com (M.U.); aturkeremre@yahoo.com.tr (T.E.); 3 Department of Cardiology, University of Health Sciences, Bursa City Training and Research Hospital, 16250 Bursa, Türkiye; selvi35byih@hotmail.com; 4 Department of Radiology, University of Health Sciences, Bursa City Training and Research Hospital, 16250 Bursa, Türkiye; drfurkanurfali@gmail.com (F.E.U.); sbco.ege.sb@gmail.com (S.B.); 5 Department of Internal Medicine, University of Health Sciences, Bursa City Hospital, 16250 Bursa, Türkiye; dogackoruk17@gmail.com

**Keywords:** peritoneal dialysis, cognitive impairment, intracranial arterial calcification, chronic kidney disease, valvular calcification, residual renal function

## Abstract

**Background:** Cognitive impairment is a common and clinically significant complication in patients undergoing peritoneal dialysis, a vulnerable population with multiple comorbidities. However, its underlying determinants remain incompletely understood. Vascular calcification, as a marker of systemic vascular pathology, has been suggested to be associated with cognitive dysfunction. This study aimed to evaluate the relationship between intracranial and valvular calcification and cognitive dysfunction in peritoneal dialysis patients. **Methods:** This single-center cross-sectional study included patients receiving peritoneal dialysis. Cognitive function was assessed using the Montreal Cognitive Assessment (MoCA). Intracranial and valvular calcifications were determined based on imaging findings. Clinical, demographic, and laboratory parameters were analyzed. Logistic regression analysis was used to identify factors associated with cognitive dysfunction, and receiver operating characteristic (ROC) analysis was performed to assess discriminative performance. **Results:** A total of 95 patients were included. Patients with cognitive dysfunction were older and more frequently female. In univariate analysis, age, female sex, and mitral valve calcification were significantly associated with cognitive dysfunction. After multivariable adjustment, only age (OR: 1.06, *p* = 0.02) and female sex (OR: 3.4, *p* = 0.03) remained independently associated. ROC analysis showed limited discriminative performance (AUC: 0.63). **Conclusions:** Calcification parameters were associated with cognitive dysfunction in unadjusted analyses but did not remain independent predictors after adjustment. Age was the strongest factor associated with cognitive dysfunction, while female sex was also independently associated with increased risk. These findings suggest that calcification burden may reflect cumulative vascular aging and comorbidity rather than serving as a standalone predictor of cognitive impairment.

## 1. Introduction

Cognitive impairment is increasingly recognized as a common and clinically significant complication in patients with chronic kidney disease (CKD), particularly among those receiving dialysis [1,2]. Cognitive dysfunction is particularly relevant in patients undergoing peritoneal dialysis (PD), as this treatment modality requires active patient participation, self-management, and adherence to complex home-based procedures. Impaired cognitive function may adversely affect treatment adherence, quality of life, and overall prognosis in this population [3,4].

Cognitive function can be assessed using several screening tools, among which the Mini-Mental State Examination (MMSE) and the Montreal Cognitive Assessment (MoCA) are the most widely used. While MMSE is commonly employed for general cognitive screening and remains a historically validated instrument, MoCA is considered more sensitive for detecting mild cognitive impairment, particularly in executive and visuospatial domains [5]. Therefore, in the present study, both tools were administered, with MoCA used as the primary tool for defining cognitive dysfunction.

Vascular calcification is a well-established complication of CKD and is closely linked to disturbances in mineral metabolism, chronic inflammation, and cardiovascular risk burden [6,7]. In patients with CKD, calcification may involve multiple vascular and valvular sites, including cardiac valves and intracranial arteries, and may reflect systemic vascular pathology rather than an isolated local process.

Valvular calcification, particularly involving the aortic and mitral valves, has been associated with increased cardiovascular morbidity and mortality in dialysis patients [8]. In dialysis patients, the reported prevalence of aortic and mitral valve calcification varies widely, and calcification may develop earlier than in the general population [9,10].

Intracranial vascular calcification has also been associated with cerebral small vessel disease, impaired cerebral perfusion, and cognitive decline in previous studies [11]. However, the relationship between calcification and cognitive dysfunction is complex and may be strongly influenced by shared cardiovascular risk factors, including age, diabetes mellitus, and hypertension [12]. In addition, inflammation, anemia, malnutrition, and mineral metabolism abnormalities may contribute to both vascular calcification and impaired cognitive function in patients with CKD [13,14,15]. These overlapping mechanisms suggest that vascular and valvular calcifications may act as markers of cumulative vascular and metabolic burden rather than direct determinants of cognitive impairment.

Previous studies have suggested that dialysis modality may influence cognitive outcomes. A recent systematic review and meta-analysis reported a lower risk of cognitive impairment in patients undergoing PD compared with those receiving hemodialysis [16]. Potential explanations include better preservation of residual renal function, greater hemodynamic stability, and differences in comorbidity or vascular burden. Nevertheless, cognitive dysfunction remains clinically important in PD patients, and the factors associated with impaired cognition in this population require further clarification.

Despite increasing interest in cognitive impairment among dialysis patients, the relationship between vascular or valvular calcification and cognitive dysfunction in patients undergoing PD remains insufficiently explored. In particular, it is unclear whether structural calcification findings provide independent information beyond traditional clinical and dialysis-related risk factors.

Therefore, this study aimed to evaluate the relationship between intracranial and valvular calcifications and cognitive dysfunction in patients undergoing PD. We also sought to identify the main clinical factors associated with cognitive dysfunction and to determine whether calcification parameters remained independently associated after adjustment for relevant confounders.

## 2. Materials and Methods

### 2.1. Study Design and Population

This single-center cross-sectional observational study was conducted in the Per-itoneal Dialysis Unit of the Department of Nephrology at Bursa City Hospital between 22 January 2025 and 22 July 2025. The study population consisted of adult patients actively receiving peritoneal dialysis during the study period. Among 125 patients actively receiving peritoneal dialysis, 95 patients met the inclusion criteria and were included in the final analysis. Echocardiographic da-ta were available in 88 patients, and analyses involving valvular calcification were conducted in this subgroup.

#### 2.1.1. Inclusion Criteria

Patients were eligible for inclusion if they met the following criteria:

- Age between 18 and 80 years;
- Undergoing peritoneal dialysis for at least 3 months;
- Brain computed tomography performed within the last 3 months;
- Availability of complete clinical, laboratory, and cognitive assessment data.
- Echocardiographic evaluation was available in 88 patients; therefore, analyses involving valvular calcification were restricted to this subgroup.

#### 2.1.2. Exclusion Criteria

- Duration of peritoneal dialysis <3 months,
- A diagnosis of dementia or Alzheimer’s disease,
- Illiteracy,
- History of intracranial hemorrhage,
- Severe depression or major psychiatric disorders requiring treatment.

### 2.2. Data Collection

Clinical and demographic data were recorded at the study visit, and relevant laboratory, imaging, and dialysis-related data were obtained from patient records using values closest to the date of cognitive assessment. These data included age, sex, dialysis duration time, primary renal disease, comorbid conditions including diabetes mellitus, hypertension, and coronary artery disease, and relevant medical history.

All participants provided written informed consent prior to enrollment.

Information regarding medications was also recorded, including phosphate binders (calcium-based and non–calcium-based), vitamin D receptor activators (calcitriol or alfacalcidol), and other relevant treatments.

Laboratory parameters were obtained from fasting venous blood samples collected in the morning (between 07:00 and 09:00 AM) under standardized conditions. All measurements were performed in the same laboratory using standard automated methods. The recorded laboratory variables included hemoglobin, serum creatinine, blood urea nitrogen, calcium, phosphorus, albumin, parathyroid hormone (PTH), 25-hydroxyvitamin D, lipid profile, and other routine biochemical parameters.

Dialysis adequacy parameters, including Kt/V and peritoneal equilibration test (PET) characteristics, were recorded. The calcium–phosphorus product (Ca × P) was calculated as the product of serum calcium and phosphorus levels, and values >55 mg^2^/dL^2^ were considered elevated. For each patient, laboratory values closest to the date of imaging and cognitive assessment were used in the analysis.

### 2.3. Assessment of Cognitive Function

Cognitive function was evaluated using both the Mini-Mental State Examination (MMSE) and the Montreal Cognitive Assessment (MoCA). The MMSE was used as a general cognitive screening tool, whereas the MoCA was used as the primary tool for defining cognitive dysfunction because of its greater sensitivity for mild cognitive impairment, particularly in executive and visuospatial domains [5]. The Montreal Cognitive Assessment was administered according to the official instructions available from the MoCA website (https://mocacognition.com/; accessed on 22 January 2026). Information regarding the Mini-Mental State Examination was accessed from the official PAR website (https://www.parinc.com/; accessed on 18 April 2026). Both tests have a score range of 0–30, with lower scores indicating worse cognitive performance. In the present study, cognitive dysfunction was defined as a MoCA score <22 [17]. MMSE scores were analyzed as complementary cognitive performance measures but were not used as the primary criterion for defining cognitive dysfunction.

### 2.4. Assessment of Intracranial Arterial Calcification

Intracranial arterial calcification was evaluated using non-contrast brain computed tomography performed within the previous 3 months. All imaging studies were reviewed by an experienced radiologist who was blinded to the clinical and laboratory data. Brain computed tomography images were obtained using a SOMATOM Definition AS CT scanner (Siemens Healthineers, Erlangen, Germany).

Calcification burden was assessed using the Agatston scoring method [18]. Because the number of patients with higher Agatston scores was limited, subgroup analysis according to calcification severity was not considered statistically appropriate. Therefore, for the primary statistical analyses, intracranial arterial calcification was categorized as absent or present, defined as an Agatston score of 0 or >0, respectively. This dichotomization was performed to improve statistical robustness and avoid unstable estimates in sparsely populated higher-score categories.

### 2.5. Assessment of Cardiac Valvular Calcification

Echocardiographic data were analyzed in patients with available data (*n* = 88). Echocardiographic examinations were performed using a Vivid ultrasound system (GE HealthCare, Chicago, IL, USA). Echocardiographic examinations performed within the previous 3 months were reviewed. Cardiac valvular calcification was assessed for the aortic and mitral valves using standard echocardiographic criteria. Valve calcification was defined as the presence of bright echoes >1 mm on one or more cusps of the aortic valve, mitral valve, or mitral annulus [19].

### 2.6. Ethics Statement

The study was conducted in accordance with the Declaration of Helsinki and approved by the Ethics Committee of Bursa City Hospital (date: 22 January 2025, Approval no: 2025-2/1). Written informed consent was obtained from all participants included in the study.

### 2.7. Data Analysis

All statistical analyses were performed using IBM SPSS software version 25.0 (IBM Corp., Armonk, NY, USA). Continuous variables were expressed as mean ± standard deviation or median (interquartile range), depending on the distribution of the data, while categorical variables were presented as frequencies and percentages. Normality of data distribution was assessed using the Kolmogorov–Smirnov test.

Comparisons between groups were performed using the independent samples t-test or Mann–Whitney U test for continuous variables, and the chi-square test or Fisher’s exact test for categorical variables, as appropriate. Patients were categorized according to the presence of cognitive dysfunction and calcification status.

Univariate and multivariate logistic regression analyses were conducted to identify factors associated with cognitive dysfunction. To reduce the risk of model overfitting due to the limited sample size, the multivariable logistic regression model was restricted to clinically relevant variables and variables associated with cognitive dysfunction in univariate analyses. Age, sex, and mitral valve calcification were included in the primary adjusted model. Additional variables were evaluated in exploratory analyses.

Multicollinearity was assessed using the variance inflation factor (VIF), and no significant collinearity was observed. Adjusted odds ratios (ORs) with 95% confidence intervals (CIs) were calculated. A two-tailed *p*-value < 0.05 was considered statistically significant.

Receiver operating characteristic (ROC) curve analysis was performed as an exploratory analysis to assess the discriminative performance of relevant parameters. Because the discriminative ability was limited, ROC-derived cut-off values were interpreted cautiously and were not considered clinically definitive.

## 3. Results

### 3.1. Baseline Characteristics

A total of 95 patients were included in the study. The mean age of the cohort was 57 ± 14 years, and 65% of the patients were male. Cognitive dysfunction, defined as a MoCA score <22, was present in 34 patients (35.8%). Echocardiographic data were available in 88 patients, and analyses involving valvular calcification were conducted in this subgroup (Table 1).

Patients with intracranial arterial calcification were significantly older than those without intracranial arterial calcification (61 ± 14 vs. 55 ± 14 years, *p* = 0.04). Similarly, patients with cognitive dysfunction were older than those without cognitive dysfunction (63 ± 12 vs. 54 ± 14 years, *p* = 0.007).

Sex distribution differed significantly according to cognitive dysfunction status, with a higher proportion of female patients among those with cognitive dysfunction (*p* = 0.03). In contrast, sex distribution did not differ according to intracranial arterial calcification status (*p* = 0.78). The prevalence of diabetes mellitus, hypertension, and coronary artery disease was comparable between groups (all *p* > 0.05).

### 3.2. Calcification Findings

Because few patients had high Agatston scores, intracranial arterial calcification was analyzed as a binary variable in the primary analyses. Mitral valve calcification was more frequent in patients with intracranial arterial calcification than in those without intracranial arterial calcification (66% vs. 43%, *p* = 0.03). Mitral valve calcification was also more frequent in patients with cognitive dysfunction than in those without cognitive dysfunction (69% vs. 45%, *p* = 0.03). In contrast, aortic valve calcification did not differ significantly according to either intracranial arterial calcification status or cognitive dysfunction status (all *p* > 0.05).

### 3.3. Laboratory and Dialysis-Related Parameters

No significant differences were observed between groups in mineral metabolism parameters, including serum calcium, phosphorus, parathyroid hormone, 25-hydroxyvitamin D, and calcium–phosphorus product (all *p* > 0.05). Serum glucose levels tended to be higher in patients with cognitive dysfunction than in those without cognitive dysfunction (135 ± 65 vs. 113 ± 44 mg/dL, *p* = 0.06). Albumin levels were lower in the cognitive dysfunction group, although this difference did not reach statistical significance (33.9 ± 7.8 vs. 36.9 ± 7.1 g/L, *p* = 0.06). Inflammatory and hematological parameters, including white blood cell count, hemoglobin, iron parameters, and ferritin, were comparable between groups.

Dialysis-related variables, including PD modality, Kt/V, ultrafiltration volume, and peritoneal transport status, did not differ significantly according to cognitive dysfunction or intracranial arterial calcification status. However, residual urine volume was significantly lower in patients with cognitive dysfunction than in those without cognitive dysfunction (840 ± 608 vs. 1227 ± 841 mL/day, *p* = 0.02).

Medication use, including phosphate binders, cinacalcet, iron therapy, erythropoietin, and vitamin D receptor activators, is summarized in Supplementary Table S1. These treatment-related findings were considered exploratory and were not included in the primary interpretation of cognitive outcomes.

### 3.4. Cognitive Function

Cognitive test scores did not differ significantly according to intracranial arterial calcification status. Patients classified as having cognitive dysfunction had lower MMSE and MoCA scores than those without cognitive dysfunction. The mean MMSE score was 21.4 ± 5.7 in patients with cognitive dysfunction and 25.8 ± 2.5 in those without cognitive dysfunction (*p* = 0.001). The mean MoCA score was also lower in patients with cognitive dysfunction than in those without cognitive dysfunction (17.4 ± 4.4 vs. 25.6 ± 2.1, *p* < 0.001), as expected based on the MoCA-based definition of cognitive dysfunction (Table 2).

#### 3.4.1. Regression Analysis

In univariate logistic regression analysis, age (OR: 1.05, 95% CI: 1.01–1.09, *p* = 0.009), female sex (OR: 2.8, 95% CI: 1.1–6.8, *p* = 0.02), and mitral valve calcification (OR: 2.7, 95% CI: 1.1–6.8, *p* = 0.03) were associated with cognitive dysfunction. Residual urine volume <950 mL/day showed borderline significance (OR: 2.36, 95% CI: 1.00–5.56, *p* = 0.05), while albumin showed a non-significant trend (OR: 0.99, 95% CI: 0.98–1.00, *p* = 0.07) (Table 3).

In this adjusted model, age and female sex remained independently associated with cognitive dysfunction, whereas mitral valve calcification was no longer independently associated.

#### 3.4.2. Receiver Operating Characteristic (Roc) Analysis

**Figure 1:**
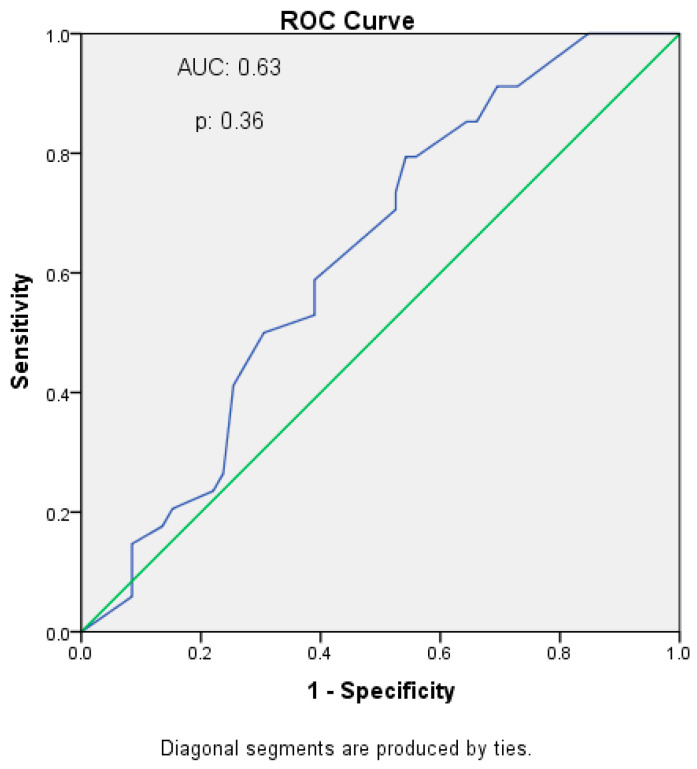
Receiver operating characteristic curve of residual urine volume for cognitive dysfunction The blue curve indicates the discriminative performance of residual urine volume, and the green diagonal line represents the no-discrimination reference line. A cut-off value of 950 mL/day yielded 60% sensitivity and 61% specificity, with limited discriminative performance (AUC: 0.63).

Receiver operating characteristic analysis was performed as an exploratory analysis to evaluate the discriminative performance of residual urine volume for cognitive dysfunction. Residual urine volume demonstrated limited and statistically non-significant discriminative ability, with an AUC of 0.63 (*p* = 0.36). A cut-off value of 950 mL/day yielded a sensitivity of 60% and a specificity of 61%; however, this threshold was considered exploratory and was not regarded as clinically definitive (Figure 1).

## 4. Discussion

In this single-center cross-sectional study of patients undergoing peritoneal dialysis, intracranial arterial and valvular calcifications were associated with cognitive dysfunction in unadjusted analyses; however, these associations did not remain independent after multivariable adjustment. Age emerged as the most consistent factor associated with cognitive dysfunction, while female sex also remained independently associated in the adjusted model. These findings suggest that calcification burden may reflect cumulative vascular aging, biological vulnerability, and comorbidity burden rather than acting as an independent determinant of cognitive dysfunction. In this context, vascular calcification may overlap with the concept of vascular frailty, reflecting long-term exposure to cardiovascular, metabolic, and inflammatory stressors [20].

Cognitive dysfunction is common in patients with CKD and dialysis and is considered a multifactorial condition involving vascular injury, uremic toxin accumulation, chronic inflammation, oxidative stress, and impaired cerebral perfusion [1,2,21]. In patients undergoing PD, cognitive dysfunction is particularly relevant because treatment requires active patient participation, self-management, and adherence.

In our study, older age was significantly associated with cognitive dysfunction, consistent with previous evidence demonstrating that advancing age is a major determinant of both vascular calcification and cognitive decline in patients with CKD [21]. Cognitive impairment may adversely affect treatment adherence, self-management, and quality of life and has also been associated with increased morbidity and mortality in patients with CKD [22,23]. Therefore, careful cognitive assessment may be particularly important in older patients undergoing PD.

In the present study, mitral valve calcification was associated with both intracranial arterial calcification and cognitive dysfunction in unadjusted analyses. However, this association did not persist after multivariable adjustment. Therefore, mitral valve calcification should be interpreted cautiously as a marker of cumulative vascular burden rather than as an independent determinant of cognitive dysfunction. Shared mechanisms such as endothelial dysfunction, arterial stiffness, and impaired cerebral perfusion may partly explain the observed unadjusted association, but causal inference cannot be made from the present cross-sectional data [24,25]. Routine echocardiographic findings may provide additional information on vascular burden, but their role in cognitive risk stratification requires confirmation in larger prospective studies.

The use of both MMSE and MoCA allowed for a more comprehensive evaluation of cognitive function. Although MMSE is widely used for general cognitive screening, it may underestimate mild cognitive impairment, particularly in executive and visuospatial domains. In contrast, MoCA is considered more sensitive in detecting subtle cognitive deficits, which are frequently observed in patients with CKD [17,26]. The parallel reduction in MMSE scores among patients classified as cognitively impaired by MoCA supports the consistency of the cognitive assessment.

Beyond structural markers such as valvular calcification, functional parameters may also contribute to cognitive outcomes in dialysis patients. In this context, residual renal function (RRF) was associated with cognitive status in unadjusted analyses in our study, as reflected by lower residual urine volume in patients with cognitive dysfunction.

RRF is a well-established determinant of survival and clinical outcomes in patients undergoing dialysis and has been associated with reduced comorbidity and improved quality of life [27]. Although RRF typically declines after dialysis initiation, peritoneal dialysis has been reported to better preserve residual kidney function compared with hemodialysis; however, this decline is influenced by multiple patient- and treatment-related factors [28].

In our study, lower residual urine volume was observed in patients with cognitive dysfunction, suggesting a possible relationship between preserved residual renal function and cognitive status. However, this finding should be interpreted cautiously, as residual urine volume showed limited discriminative performance and did not remain independently associated with cognitive dysfunction after adjustment. Therefore, RRF alone should not be considered a standalone marker of cognitive dysfunction.

The potential relationship between RRF and cognition is biologically plausible. Unlike conventional dialysis adequacy parameters such as Kt/V, which primarily reflect small solute clearance, RRF contributes to the continuous removal of middle molecules and protein-bound uremic toxins [27]. These substances have been implicated in systemic inflammation, endothelial dysfunction, and neurotoxicity in patients with CKD. In addition, preserved RRF may be associated with better volume control and more stable hemodynamic status, which could indirectly support cerebral perfusion and cognitive performance [29]. Nevertheless, given the cross-sectional design of the present study, these mechanisms remain hypothetical and should be confirmed in prospective studies.

In our study, peritoneal transport characteristics, as assessed by PET, were not associated with either cognitive dysfunction or intracranial arterial vascular calcification. Although PET reflects peritoneal membrane transport properties, it may not fully capture systemic factors such as vascular injury, endothelial dysfunction, and accumulation of neurotoxic substances, which are more directly involved in cognitive decline. This finding further reinforces the concept that conventional dialysis-related parameters may be insufficient to explain the multifactorial nature of cognitive dysfunction in patients undergoing peritoneal dialysis.

Laboratory parameters related to mineral metabolism, including calcium, phosphorus, parathyroid hormone, calcium–phosphorus product, 25-hydroxyvitamin D, and albumin, were not associated with either intracranial arterial calcification or cognitive dysfunction. This may suggest that single time-point biochemical measurements do not fully reflect the cumulative metabolic, inflammatory, and vascular burden in patients with CKD. Therefore, the absence of significant associations should be interpreted cautiously rather than as evidence that these pathways are not involved.

We observed a lower prevalence of mitral valve calcification among patients receiving vitamin D receptor activators (Supplementary Table S1). This finding should be interpreted cautiously, as treatment allocation was not randomized and may reflect differences in patient management, mineral metabolism status, or underlying disease severity. Nevertheless, this observation is biologically plausible, since vitamin D is involved in calcium–phosphorus homeostasis and may influence vascular smooth muscle cell differentiation and calcification-related pathways [30]. Experimental studies have also suggested that vitamin D receptor activation may modulate vascular calcification processes, including effects on Klotho and osteopontin expression under high-phosphate conditions [31]. However, given the cross-sectional design of the present study, no causal inference can be made, and this finding requires confirmation in larger prospective studies.

Vascular calcification is a progressive and time-dependent process driven by prolonged exposure to multiple interrelated factors, including chronic inflammation, oxidative stress, and dysregulated mineral metabolism [7]. Accordingly, current laboratory values may fail to capture the historical metabolic burden contributing to calcification. Similarly, cognitive dysfunction in dialysis patients is complex and multifactorial. The absence of significant associations between routine laboratory parameters and cognitive outcomes in our study suggests that isolated biochemical measurements may be insufficient to explain neurocognitive impairment in this population.

After adjustment, age and female sex remained independently associated with cognitive dysfunction. The association with age is consistent with the well-established role of aging in both vascular pathology and cognitive decline. The finding regarding female sex should be interpreted cautiously, as sex-related differences in cognitive performance may be influenced by age distribution, educational background, comorbidity burden, and other unmeasured social or biological factors [32,33].

ROC analysis demonstrated limited and statistically non-significant discriminative performance of residual urine volume for cognitive dysfunction. This finding indicates that residual urine volume alone is insufficient for identifying cognitive dysfunction and further supports the multifactorial nature of cognitive impairment in patients undergoing peritoneal dialysis.

The lack of an independent association between calcification and cognitive dysfunction may be partly explained by the confounding effect of age, as both calcification burden and cognitive decline increase with advancing age [21]. This interpretation is also consistent with the concept that calcification burden may reflect vascular aging rather than chronological age alone. A recent study introduced the concept of coronary arterial age, emphasizing that vascular aging may progress faster or slower than chronological aging [34]. Therefore, future studies should consider whether calcification burden is disproportionate for chronological age and dialysis status, which may help clarify its relationship with cognitive dysfunction in patients undergoing peritoneal dialysis.

This study has several limitations. First, its cross-sectional design precludes causal inference and does not allow temporal or prognostic interpretation. Longitudinal outcomes, including mortality, cardiovascular events, stroke, and cognitive decline over time, were not assessed. Second, the study was conducted in a single center with a relatively small sample size, which limited the number of variables that could be included in the multivariable model and increased the possibility of residual confounding. Third, cognitive function was assessed using screening tools rather than comprehensive neuropsychological testing. Fourth, educational level and socioeconomic status were not systematically assessed, although both may influence cognitive test performance. Fifth, echocardiographic data were available in 88 patients, and analyses involving valvular calcification were therefore restricted to this subgroup. Finally, coronary artery and thoracic aortic calcifications were not assessed; therefore, the full burden of multi-site vascular calcification could not be evaluated.

Future studies incorporating coronary, thoracic aortic, intracranial, and valvular calcification assessments, together with longitudinal cognitive and clinical outcomes, are needed to better clarify the relationship between systemic calcification burden and cognitive dysfunction in peritoneal dialysis patients.

In conclusion, intracranial arterial and valvular calcifications were associated with cognitive dysfunction in unadjusted analyses but were not independent predictors after multivariable adjustment. Age was the most consistent factor associated with cognitive dysfunction, while female sex also remained independently associated in the adjusted model. These findings suggest that calcification burden may reflect cumulative vascular and comorbidity burden rather than directly determining cognitive dysfunction in patients undergoing peritoneal dialysis. Larger prospective studies incorporating educational, socioeconomic, frailty, and longitudinal cognitive assessments are needed.

## Figures and Tables

**Table 1:** Baseline clinical, laboratory, and dialysis-related characteristics according to intracranial arterial calcification and cognitive dysfunction status.

Variable	Overall (*n* = 95)	No Intracranial Arterial Calcification (*n* = 50)	Intracranial Arterial Calcification (*n* = 45)	No Cognitive Dysfunction (*n* = 61)	Cognitive Dysfunction (*n* = 34)	*p* Value (p1/p2)
Age	57 ± 14	55 ± 14	61 ± 14	54 ± 14	63 ± 12	0.04/0.007
Male, *n* (%)	62 (65)	32 (64)	30 (67)	45 (74)	17 (50)	0.78/0.03
DM, *n* (%)	35 (37)	19 (38)	16 (36)	20 (33)	15 (44)	0.80/0.27
HT, *n* (%)	93 (98)	50 (100)	43 (96)	59 (97)	34 (100)	0.42/0.75
Coronary artery disease, *n* (%)	31 (33)	12 (24)	19 (42)	16 (26)	15 (44)	0.06/0.11
Aortic valve calcification †, *n* (%)	51 (58)	26 (55)	25 (61)	33 (59)	18 (56)	0.59/0.81
Mitral valve calcification †, *n* (%)	47 (53)	20 (43)	27 (66)	25 (45)	22 (69)	0.03/0.03
Vitamin D (ng/mL)	9.4 ± 7.5	10.2 ± 7.9	9.3 ± 8.2	10.1 ± 8.3	9.1 ± 7.4	0.59/0.54
PTH (pg/mL)	322 ± 213	332 ± 224	321 ± 243	332 ± 237	319 ± 228	0.82/0.79
Phosphorus (mg/dL)	4.8 ± 1.3	4.6 ± 1.3	4.9 ± 1.3	4.9 ± 1.3	4.6 ± 1.2	0.24/0.36
Calcium (mg/dL)	8.9 ± 1.1	8.8 ± 1.3	9.1 ± 0.7	9.1 ± 0.6	9.0 ± 0.7	0.14/0.53
Ca × P (mg^2^/dL^2^)	43.9 ± 12.2	42.1 ± 12.7	45.0 ± 11.5	44.7 ± 12.8	41.3 ± 10.8	0.25/0.18
Ca × P > 55 mg^2^/dL^2^, *n* (%)	18 (19)	7 (14)	11 (24)	13 (21)	5 (15)	0.19/0.61
Glucose (mg/dL)	120 ± 52	124 ± 62	117 ± 41	113 ± 44	135 ± 65	0.54/0.06
LDL cholesterol (mg/dL)	117 ± 40	115 ± 39	118 ± 41	119 ± 39	114 ± 42	0.68/0.53
Urea (mg/dL)	101 ± 34.7	98.6 ± 36.3	101.6 ± 32.8	103.5 ± 36.4	93.8 ± 30.5	0.69/0.19
Creatinine (mg/dL)	5.9 ± 3.1	5.7 ± 2.9	5.9 ± 3.3	5.8 ± 2.9	5.9 ± 3.3	0.74/0.87
Sodium (mmol/L)	138 ± 4	138 ± 3	138 ± 4	138 ± 3	138 ± 4	0.92/0.95
Potassium (mmol/L)	4.5 ± 0.8	4.4 ± 0.6	4.5 ± 0.9	4.5 ± 0.8	4.4 ± 0.7	0.62/0.63
Uric acid (mg/dL)	5.5 ± 1.6	5.4 ± 1.6	5.5 ± 1.5	5.5 ± 1.6	5.3 ± 1.4	0.65/0.62
Albumin (g/L)	36.0 ± 7.4	36.0 ± 7.7	35.7 ± 7.3	36.9 ± 7.1	33.9 ± 7.8	0.91/0.06
CAPD, *n* (%)	85 (89)	46 (92)	39 (87)	55 (90)	30 (88)	0.61/0.76
PET (low/low-average), *n* (%)	50 (53)	26 (52)	24 (53)	35 (57)	15 (44)	0.89/0.22
Kt/V, mean ± SD	2.3 ± 0.4	2.3 ± 0.4	2.3 ± 0.5	2.3 ± 0.5	2.3 ± 0.4	0.69/0.61
Residual urine (mL/day)	1070 ± 773	1142 ± 789	1020 ± 780	1227 ± 841	840 ± 608	0.46/0.02
Ultrafiltration (mL/day)	1170 ± 660	1217 ± 706	1111 ± 608	1138 ± 674	1220 ± 646	0.45/0.57
Duration of peritoneal dialysis, months	17.5 (10.2–30.0)	15.0 (8.0–29.3)	22.0 (15.0–32.0)	20.0 (13.5–32.0)	16.0 (7.0–27.5)	0.09/0.13
White blood cell count (×10^9^/L)	8.3 ± 3.1	8.4 ± 3.1	8.2 ± 2.9	8.1 ± 2.4	8.6 ± 3.9	0.69/0.51
Hemoglobin (g/dL)	10.9 ± 2.1	11.1 ± 1.7	10.7 ± 2.4	10.9 ± 2.2	10.8 ± 1.9	0.42/0.91
Iron (µg/dL)	70.8 ± 33.3	68.2 ± 35.8	73.4 ± 29.9	70.2 ± 33.1	71.6 ± 33.5	0.45/0.84
TIBC (µg/dL)	253 ± 49	251 ± 46	253 ± 56	259 ± 52	241 ± 48	0.88/0.10
Ferritin (ng/mL)	519 ± 470	551 ± 457	489 ± 488	460 ± 349	634 ± 626	0.53/0.08

*p1: comparison between patients with and without intracranial arterial calcification; p2: comparison between patients with and without cognitive dysfunction. Data are presented as mean ± standard deviation, median (interquartile range), or n (%)*. Cognitive dysfunction was defined as a MoCA score <22. † Echocardiographic variables were analyzed in the subgroup with available echocardiographic data (*n* = 88). Denominators for echocardiographic variables were *n* = 47 and *n* = 41 for patients without and with intracranial arterial calcification, and *n* = 56 and *n* = 32 for patients without and with cognitive dysfunction, respectively. Abbreviations: DM, diabetes mellitus; HT, hypertension; PTH, parathyroid hormone; Ca × P, calcium–phosphorus product; TIBC, total iron-binding capacity; LDL, low-density lipoprotein; CAPD, continuous ambulatory peritoneal dialysis; PET, peritoneal equilibration test.

**Table 2:** Cognitive assessment scores according to intracranial arterial calcification and cognitive dysfunction status.

Variable	No Intracranial Arterial Calcification (*n* = 50)	Intracranial Arterial Calcification (*n* = 45)	*p* Value	No Cognitive Dysfunction (*n* = 61)	Cognitive Dysfunction (*n* = 34)	*p* Value
MMSE score	24.7 ± 4.4	23.8 ± 4.6	0.34	25.8 ± 2.5	21.4 ± 5.7	0.001
MoCA score	23.3 ± 4.7	22.1 ± 5.4	0.25	25.6 ± 2.1	17.4 ± 4.4	<0.001

Data are presented as mean ± standard deviation. Cognitive dysfunction was defined as a MoCA score <22. Comparisons between groups were performed using Student’s *t*-test or Mann–Whitney U test, as appropriate. MMSE, Mini-Mental State Examination; MoCA, Montreal Cognitive Assessment.

**Table 3:** Logistic regression analysis of factors associated with cognitive dysfunction.

Variable	Univariate OR (95% CI)	*p* Value	Multivariable OR (95% CI)	*p* Value
Age	1.05 (1.01–1.09)	0.009	1.06 (1.00–1.10)	0.02
Female sex	2.8 (1.1–6.8)	0.02	3.4 (1.1–10.3)	0.03
Mitral valve calcification	2.7 (1.1–6.8)	0.03	1.7 (0.6–4.9)	0.29
Albumin	0.99 (0.98–1.00)	0.07	-	-
Residual urine <950 mL/day	2.36 (1.00–5.56)	0.05	-	-

OR, odds ratio; CI, confidence interval. Cognitive dysfunction was defined as a MoCA score <22. To reduce the risk of overfitting due to the limited sample size, the multivariable logistic regression model was restricted to age, sex, and mitral valve calcification. Multicollinearity was assessed using the variance inflation factor, and no significant collinearity was detected. A two-tailed *p* value < 0.05 was considered statistically significant.

## Data Availability

The data that support the findings of this study are available from the corresponding author upon reasonable request.

## Supplementary Materials

The following supporting information can be downloaded at: https://www.mdpi.com/article/10.3390/jcm15103635/s1, Table S1. Association of clinical characteristics, laboratory parameters, and medication use with mitral valvular calcification in patients undergoing peritoneal dialysis. Table S2. Association of clinical characteristics, laboratory parameters, and medication use with aortic valvular calcification in patients undergoing peritoneal dialysis.
