# Supplementary material for: Cognitive Dysfunction and Vascular/Valvular Calcification in Patients Undergoing Peritoneal Dialysis: A Cross-Sectional Study
Source: J Clin Med. 2026 May 9;15(10):3635. doi: 10.3390/jcm15103635 (PMC13207282; doi:10.3390/jcm15103635)
Supplement: Supplementary file 1 [file jcm-15-03635-s001.zip › jcm-4299005-supplementary.pdf]

***Supplementary Table 1**. Association of clinical characteristics, laboratory parameters, and medication use with mitral valvular calcification in patients undergoing peritoneal dialysis*

	Overall n=88	No mitral valvular calcification n=41	Mitral valvular calcification =47	P value
Age	57±14	54±13	60±15	0.04
Male, n (%)	57(65)	30(73)	27(57)	0.12
Diabetes mellitus, n (%)	31(35)	12(29)	19(40)	0.27
Hypertension, n (%)	86(98)	40(98)	46(98)	0.92
Coronary artery disease, n (%)	27(31)	8 (20)	19(40)	**0.03**
Vascular calcification, n (%)	41(47)	14(34)	27(57)	**0.03**
Cognitive dysfunction, MoCA, n (%)	32(36)	10(24)	22(47)	**0.03**
Phosphate binder use, n (%)	61(69)	26(63)	35(74)	0.26
Vitamin D therapy, n (%)	67(76)	36(88)	31(66)	0.02
Cinacalcet use, n (%)	2(2.3)	1(2)	1(2)	0.92
Erythropoietin use, n (%)	61(69)	29(71)	32(68)	0.79
Hemoglobin (g/dL)	10.9±2.1	11.1±2.6	10.9±1.7	0.77
White blood cell ($\times 10^9$/L)	8.3±3.1	8.4±2.7	8.2±3.3	0.79
Vitamin D (ng/mL)	9.9±8.3	11.1±8.8	8.9±7.7	0.18
PTH (pg/mL)	322±213	347±215	313±246	0.49
Calcium (mg/dL)	8.9±1.1	8.9±0.7	9.1±0.6	0.39
Ca × P (mg^2/dL^2)	43.7±12.0	43.9±11.8	43.4±12.2	0.97
Ca × P >55 mg^2/dL^2, n (%)	17(19)	8(20)	9(19)	0.96
Glucose (mg/dL)	119±53	115±45	123±59	0.49
LDL cholesterol (mg/dL)	118±40	116±37	119±43	0.64
Urea (mg/dL)	101.5±32.8	101.3±32.8	101.6±33.3	0.95
Creatinine (mg/dL) |	5.78±3.10	6.05±3.02	5.54±3.18	0.44
Sodium (mmol/L)	138±4	138±4	138±4	0.50
Potassium (mmol/L)	4.5±0.8	4.5±0.8	4.5±0.6	0.87
Uric acid (mg/dL)	5.6±1.4	5.7±1.1	5.5±1.6	0.33
Albumin (g/L)	36.1±7.6	36.0±8.1	36.0±7.02	0.64
CAPD, n (%)	78(88)	35(85)	43(92)	0.57
Residual urine (mL/day)	1068±760	1119±703	1053±835	0.69
Residual urine >950 (mL/day), n (%)	40(46)	18(44)	22(47)	0.43
Ultrafiltration (mL/day)	1184±677	1118±624	1245±716	0.39
Dialysis duration (months)	17.5(10.2-30.0)	17.0(9-28)	16.0(14-34)	0.50
PET (LL), n (%)	48(55)	26(63)	22(54)	0.87
Kt/V	2.3±0.5	2.4±0.5	2.3±0.4	0.37

Data are presented as mean ± standard deviation, median (interquartile range), or n (%). Comparisons were performed using the independent samples t-test or Mann–Whitney U test for continuous variables and the chi-square

test or Fisher's exact test for categorical variables, as appropriate. Valvular calcification was defined as the presence of mitral valvular calcification on echocardiography.
Abbreviations: PTH, parathyroid hormone; Ca × P, calcium–phosphorus product; LDL, low-density lipoprotein; CAPD, continuous ambulatory peritoneal dialysis; MoCA, Montreal Cognitive Assessment; PET (LL), peritoneal equilibration test, low/low-average transporter category.

Supplementary Table 2*. Association of clinical characteristics, laboratory parameters, and medication use with aortic valvular calcification in patients undergoing peritoneal dialysis*

	Overall n=88	No aortic valvular calcification n=37	Aortic valvular calcification n=51	P value
Age	57±14	53±14	61±14	**0.01**
Male, n (%)	57(65)	24(65)	33(65)	0.98
Diabetes mellitus, n (%)	31(35)	14(38)	17(33)	0.66
Hypertension, n (%)	86(98)	37(100)	49(96)	0.51
Coronary artery disease, n (%)	27(31)	11(30)	16(31)	0.87
Vascular calcification, n (%)	41(47)	16(43)	25(49)	0.59
Cognitive dysfunction, MoCA, n (%)	32(36)	14(38)	18(35)	0.81
Phosphate binder use, n (%)	61(69)	25(68)	36(71)	0.76
Vitamin D therapy, n (%)	67(76)	27(73)	40(78)	0.55
Cinacalcet use, n (%)	2(2.3)	0(0)	2(4)	0.50
Erythropoietin use, n (%)	61(69)	31(84)	30(59)	**0.01**
Hemoglobin (g/dL)	10.9±2.1	10.8±2.6	11.1±1.8	0.44
White blood cell ($\times 10^9$/L)	8.3±3.1	9.02±3.11	7.75±2.85	0.06
Vitamin D (ng/mL)	9.9±8.3	9.6±7.9	10.1±8.6	0.81

PTH (pg/mL)	322±213	310±239	343±228	0.52
Calcium (mg/dL)	8.9±1.1	8.9±0.6	9.1±0.6	0.34
CaXP (mg²/dL²)	43.7±12.0	44±12	43±12	0.85
Caxp> 55 (mg²/dL²), n (%)	17(19)	8(22)	9(18)	0.64
Glucose (mg/dL)	119±53	118±48	120±56	0.83
LDL cholesterol (mg/dL)	118±40	109±40	124±39	0.09
Urea (mg/dL)	101.5±32.8	95±31	106±34	0.15
Creatinine (mg/dL)	5.78±3.10	6.2±3.2	5.5±2.9	0.34
Sodium (mmol/L)	138±4	138±4	138±4	0.33
Potassium (mmol/L)	4.5±0.8	4.3±0.9	4.6±0.6	0.07
Uric acid (mg/dL)	5.6±1.4	5.5±1.3	5.6±1.4	0.70
Albumin (g/L)	36.1±7.6	34.5±10.1	37.2±5.1	0.11
CAPD, n (%)	78(88)	31(84)	47(92)	0.22
Residual urine total volume (mL/day)	1068±760	1069±591	1096±884	0.87
Residual urine >950 (mL/day), n (%)	40(46)	15(41)	25(49)	0.43
Ultrafiltration (mL/day)	1184±677	1104±596	1242±731	0.35
Dialysis duration (months)	17.5(10.2-30.0)	16.0(10-27)	18.0(11-38)	0.34
PET (LL), n (%)	48(55)	20(54)	28(55)	0.94
Kt/V	2.3±0.5	2.3±0.5	2.4±0.4	0.43

Data are presented as mean ± standard deviation, median (interquartile range), or n (%). Comparisons were performed using the independent samples t-test or Mann–Whitney U test for continuous variables and the chi-square test or Fisher's exact test for categorical variables, as appropriate. Valvular calcification was defined as the presence of aortic valvular calcification on echocardiography.

Abbreviations: PTH, parathyroid hormone; Ca × P, calcium–phosphorus product; LDL, low-density lipoprotein; CAPD, continuous ambulatory peritoneal dialysis; MoCA, Montreal Cognitive Assessment; PET (LL), peritoneal equilibration test, low/low-average transporter category.
